# 2-Aminophenylboronic acid-functionalized carbon dots show broad-spectrum antiviral activity against respiratory viruses

**DOI:** 10.1039/d6na00007j

**Published:** 2026-06-26

**Authors:** Musbahu Adam Ahmad, Tufael Ahmed, Mochamad Zakki Fahmi, Adi Idris

**Affiliations:** a Department of Chemistry, Airlangga University Surabaya 60115 Indonesia; b Centre for Immunology and Infection Control, School of Biomedical Sciences, Queensland University of Technology Kelvin Grove Brisbane QLD 4702 Australia a2.idris@qut.edu.au; c Supra Modification Nano-Micro Engineering Research Group, Airlangga University Surabaya 60115 Indonesia m.zakki.fahmi@fst.unair.ac.id

## Abstract

Respiratory viruses such as influenza A virus (IAV), respiratory syncytial virus (RSV), and human metapneumovirus (hMPV) impose a persistent global health burden, yet current antivirals are often limited by toxicity and side effects. This highlights the urgent need for broad-spectrum antiviral agents that can safely block viral infections. Carbon dots (cDots) provide an ideal scaffold for this strategy due to their ultra-small size, tuneable surface chemistry, and inherent biocompatibility. Notably, we previously demonstrated that 2-aminophenylboronic acid (APBA)-derived cDots have virus-binding capabilities by inhibiting human immunodeficiency virus (HIV)-1 entry. Herein, we synthesize APBA-functionalized cDots *via* pyrolysis, yielding cDots densely decorated with boronic acid and boroxine groups. These non-toxic APBA–cDots demonstrate potent antiviral activity against RSV, IAV, and hMPV *in vitro*. Competitive inhibition by *N*-acetylglucosamine and molecular docking analyses are consistent with a proposed boronic acid-mediated interaction with glycan-rich viral surfaces, although this mechanism has not yet been definitively proven by direct loss-of-function chemistry. Overall, these findings identify APBA–cDots as a promising antiviral nanoplatform and provide preliminary support for a glycan-targeting mechanism that warrants further validation.

## Abstract 2

Development of highly potent 2-aminophenylboronic acid-functionalized carbon dots with broad acting antiviral activity against several respiratory viruses. Graphical abstract image created with Blender via Blender Foundation.

## Graphical abstract

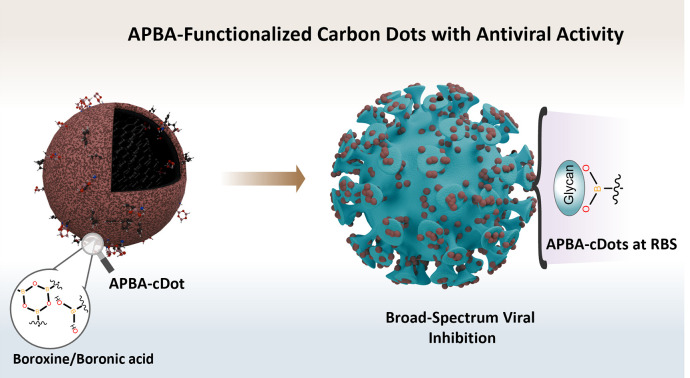

## Background

Respiratory viral infections impose a heavy global health burden, causing substantial morbidity and mortality each year. The enveloped RNA viruses such as the human respiratory syncytial virus (RSV), influenza virus (IAV), and human metapneumovirus (hMPV) are among the leading causes of acute respiratory disease in children, elderly and immunocompromised populations.^1^ These viruses produce a spectrum of illness from mild cold to severe pneumonia or acute respiratory distress syndrome (ARDS), and collectively they strain healthcare systems worldwide. Unfortunately, current therapeutics are inadequate. Effective IAV vaccines exist but are often mismatched, and antiviral drugs (*e.g.*, neuraminidase or polymerase inhibitors) are limited in number and compromised by rapid resistance.^2^ RSV has no approved antiviral treatment. The only licensed therapy (*i.e.*, ribavirin) is largely obsolete and prophylactic antibodies (palivizumab and/or nirsevimab) are restricted to high-risk infants.^3^ HMPV also has no specific antiviral therapy or vaccine and patient management remains purely supportive.^4^

Carbon dots (cDots) are emerging as a versatile nanoplatform for biomedical applications.^5^ These fluorescent carbon nanoparticles (typically <10 nm) possess a graphitic or amorphous carbon core and abundant surface functional groups. CDots are generally biocompatible and show very low toxicity *in vitro* and *in vivo*. Furthermore, cDots can be synthesized by simple methods (*i.e.*, hydrothermal or pyrolysis of organic precursors), and the resulting surface chemistry is readily modifiable. Building on these concepts, several studies have demonstrated that boronic acid-functionalized cDots can act as antiviral agents^6^ against viruses such as human coronavirus (HCoV-229E)^7^ and herpes simplex virus (HSV)^8^ by preventing their entry into cells. Notably, we previously developed 2 nm graphitic cDots by pyrolysis of citric acid (CA) and then appended phenylboronic acid groups.^9^ These 2-aminophenylboronic acid (APBA)-derived cDots potently inhibited HIV-1 cell entry by binding to its gp120 glycoprotein. Inspired by this, we hypothesized that cDots decorated with APBA would act as effective neutralizing agents against common respiratory viruses associated with seasonal outbreaks (RSV, IAV, and hMPV). Indeed, a common feature of many enveloped viruses is the heavy glycosylation of their surface proteins. The APBA ligand contains a boronic acid moiety that can bind *cis*-diols on viral glycans, as well as an amino group that may enhance binding or solubility. By functionalizing cDots with APBA, we create multivalent nanoparticles densely decorated with boronic acid and boroxine groups.

Here we report the synthesis, characterization, and antiviral evaluation of APBA–cDots. Pyrolysis of 2-APBA yields fluorescent cDots with a graphitic-like core and surfaces rich in boronic acid/boroxine functionalities. We demonstrate that these non-toxic APBA–cDots exhibit potent antiviral activity against tested respiratory viruses *in vitro*. These results establish APBA–cDots as a new antiviral nanomaterial platform that merges the structural benefits of cDots with the glycan-targeting power of boronic acids.

## Results and discussion

### Physico-chemical characterization of APBA–cDots

**Fig. 1:**
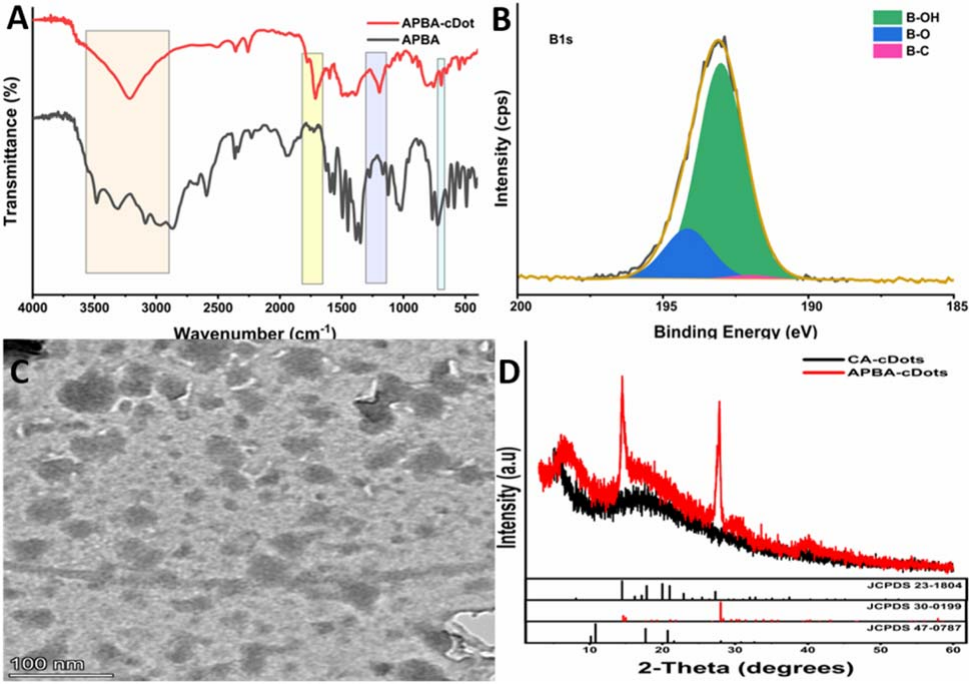
Physicochemical characterisation of APBA–cDots. (A) Fourier-transform infrared (FTIR) spectra of APBA–cDots (red line) and the free APBA precursor (black line). Key assignments include: broad O–H/N–H stretching at ∼3215 cm^−1^; C=O carboxyl at 1713 cm^−1^; C=O amide at 1597 cm^−1^; B–O and B–OH stretching at 1384 and 1190 cm^−1^, respectively; B–C stretching at 1072 cm^−1^ (shifted from the C–N stretch at 1021 cm^−1^ in free APBA, confirming covalent integration of the phenylboronic acid moiety into the carbon matrix); and a diagnostic out-of-plane ring deformation of boroxine rings (B_3_O_3_) at ∼691 cm^−1^, confirming surface dehydration and self-assembly of boronic acid groups into a boroxine-rich architecture. (B) High-resolution XPS B 1s spectrum of APBA–cDots deconvoluted into three components: a dominant peak at 193.0 eV assigned to B–OH bonds, a peak at 194.1 eV assigned to B–O bonds, and a lower-intensity peak at 192.0 eV corresponding to residual B–C bonds retained from the APBA precursor. These assignments confirm the presence of surface-accessible boronic acid and boroxine functionalities on the cDot surface. (C) Transmission electron microscopy (TEM) micrograph of APBA–cDots. Particles are well-dispersed, quasi-spherical, and uniform in size, with an average diameter of approximately 3 nm, consistent with the established size range for carbon dots (0–10 nm). Scale bar as indicated. (D) X-ray diffraction (XRD) patterns of CA–cDots (black line) and APBA–cDots (red line). The broad diffraction feature reflects the characteristic amorphous-to-graphitic carbon core structure of cDots. Additional peaks at 2*θ* = 14.5° and 28.0° in the APBA–cDot pattern match reference peaks for boric acid, confirming the presence of boron-containing surface functionalities. The shift and broadening of peaks relative to CA–cDots are consistent with successful APBA incorporation during pyrolysis synthesis.

We first characterised the APBA–cDots post-synthesis before testing their antiviral activity against a range of respiratory viruses. UV-vis analysis suggested the presence of an sp^2^ aromatic carbon skeleton and heteroatom functionalization (Fig. S1A). The APBA–cDots exhibited fluorescence upon UV light absorption. PL studies on APBA–cDots indicated an excitation-dependent emission pattern; highest intensity was achieved under 400 nm excitation (Fig. S1B). Subsequently, TEM confirmed the average diameter of these nanodots to be approximately 3 nm (Fig. 1C and S1D). This falls within the widely cited size range of cDots (0–10 nm).^9^ However, AFM analysis revealed an average height of around 16 nm likely due to cluster formation during sample preparations (Fig. S2).

As discussed in our previous report, the XRD pattern of APBA–cDots (Fig. 1D) revealed the presence of a boronic acid structure on the surface of the cDots.^10^ Interestingly, the additional peaks at 14.5° and 28.0° matched well with those of boric acid (JCPDS 30-0199).^11^ Furthermore, Raman spectroscopy (Fig. S1C) showcased D and G bands at 1359 cm^−1^ and 1596 cm^−1^, respectively. In addition, a second-order Raman band arising from overtone and/or combination appeared between 2500 and 3500 cm^−1^.^12,13,14^ Overall, the data indicate a significant level of graphitization (*I*_D_/*I*_G_ = 0.77) in APBA–cDots. While these data are consistent with a graphitic-like carbon core structure, direct lattice-resolution imaging (HRTEM) was not performed and represents a characterisation gap to be addressed in future work.

The surface chemistry and bonding states were analysed using X-ray photoelectron spectroscopy (XPS) and Fourier transform infrared (FTIR) spectroscopy (Fig. 1A). The XPS survey spectrum revealed the elements present are mainly boron and oxygen (Fig. S3). To further compare the surface charge properties of CA–cDots and APBA–cDots (Fig. S4), we measured their zeta potentials in aqueous dispersion. CA–cDots displayed a highly negative zeta potential (−55.6 mV), consistent with a carboxylate- and hydroxyl-rich surface. In contrast, APBA–cDots showed a substantially less negative zeta potential (−10.0 mV), indicating partial charge compensation and surface reorganisation upon introduction of nitrogen- and boron-containing APBA groups. This shift toward more moderate negative charge is compatible with dense APBA functionalisation and may influence colloidal stability and interactions with biological interfaces. High-resolution B 1s deconvolution identified a dominant peak at 193 eV followed by a peak at 194.1 eV (Fig. 1B) attributed to B–OH and B–O bonds, respectively.^10^ Additionally, the lowest intensity peak at 192.0 eV corresponded to B–C bonds retained from the precursor.^15,16^ As for FTIR, the broad and pointy tip peak at 3215 cm^−1^ could be attributed to O–H stretching (due to its broadness) and N–H stretching (sharp end) (Fig. 1A). The disappearance of double (H–N–H) peaks at 3484 and 3315 cm^−1^ confirms the covalent linkage of APBA during synthesis. Furthermore, the peaks at 1713, 1597, 1384, and 1190 cm^−1^ could be attributed to C=O (carboxyl), C=O (2° amide), B–O and B–OH, respectively.^10^ Importantly, the APBA–cDots spectrum exhibited a diagnostic peak around 691 cm^−1^, which is assigned to the out-of-plane ring deformation of boroxine rings (B_3_O_3_), confirming that the boronic acid groups underwent dehydration and self-assembly on the cDot surface.^17^ The disappearance of the characteristic C–N stretching peak at 1021 cm^−1^ of APBA and the emergence of new signals at 1072 cm^−1^ (B–C stretching) further confirm the covalent incorporation of the phenylboronic acid moiety into the carbon matrix. This boroxine-rich surface architecture is critical for the anticipated antiviral mechanism, providing a rigid, multivalent scaffold for diol binding. Overall, the multivalent boronic acid surface on APBA–cDots we designed here is hypothesised to bind viral envelope glycans, hinting at a glycan-targeted mechanism that could be generalized to diverse enveloped viruses. We therefore proceeded to test these cDots against a range of respiratory viral strains *in vitro*.

### APBA–cDots are not cytotoxic across a range of respiratory virus-permissive cell types

We first assessed the biocompatibility of APBA–cDots relative to unmodified CA–cDots in MDCK, LLC-MK2 and HEp-2 cell lines using a CCK-8 viability assay (Fig. S5). In all three cell types, treatment with APBA–cDots at the concentrations tested caused no significant loss of viability (relative to vehicle control) after 24 or 48 h. Cell viability remained essentially unchanged across the entire concentration range for both 24 and 48 h (Fig. S5), and there were no significant differences between APBA–cDots and CA–cDots. The lack of toxicity we observe for APBA–cDots is entirely consistent with the well-documented biocompatibility of cDots. Importantly, surface chemistry rarely undermines this safety. N-doped cDots show very low cytotoxicity across diverse mammalian lines. CDots have been shown to cause very low cytotoxicity to MDCK and HeLa cells even at high concentrations,^18^ whereas Liu *et al.*^19^ reported little to no toxicity of HepG2 cells when exposed to high amounts of cDots complexed to polyethylenimine (PEI). 4-APBA-derived CDots were essentially non-cytotoxic to both A549 and Vero cells,^20^ and we showed that carboxyboronated cDots were also non-toxic to MOLT-4 cells even at high concentrations.^9^ In all cases, the antiviral activity of our APBA–cDots came at doses far below any cytotoxic threshold (Fig. S5). Overall, APBA functionalization did not introduce host cell toxicity, confirming that these cDots can be used at antiviral doses without compromising host cell health.

### APBA–cDots potently neutralise viral infection *in vitro*

**Fig. 2:**
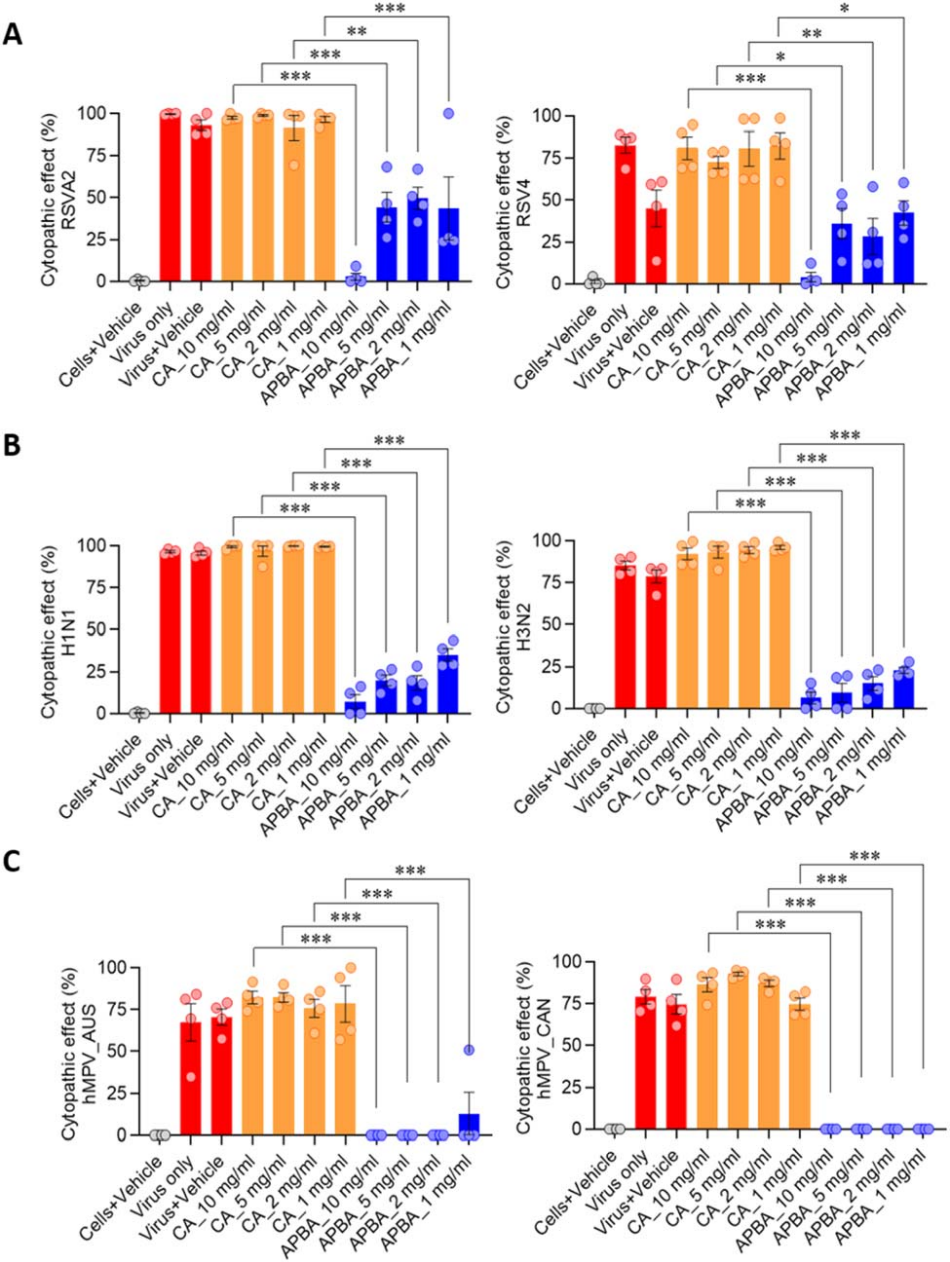
APBA–cDots potently neutralise viral infection *in vitro*. Anti-viral activity of either APBA-modified cDots (APBA) or unmodified cDots (CA) at increasing concentrations, measured as inhibition of virus-mediated cytopathic effect (CPE), against a range of viruses: (A) RSV strains RSV-A2 (left) and RSV4 (right), (B) IAV strain H1N1 (left) and H3N2 (right) and (C) HMPV strains AUS-001 (AUS) (left) and CAN-97-83 (CAN) (right). 0.1% DMSO diluted in cell media serves as the vehicle control. Data are represented by scatter plots, with bars representing mean ± SEM. Statistical significance between different groups was determined using one-way ANOVA with Tukey's post-hoc test and * denotes *p* < 0.05, ** denotes *p* < 0.01 and *** denotes *p* < 0.001.

We next evaluated APBA–cDots for antiviral efficacy against a panel of respiratory viruses. We performed co-incubation of virus with increasing concentrations of APBA–cDots or CA–cDots, followed by infection of the appropriate host cells. Antiviral activity was measured as the inhibition of virus-induced cytopathic effect (CPE) and viral immunoplaque formation after 7 days. The results show a clear, concentration-dependent neutralization of infection by APBA–cDots for all virus strains, whereas CA–cDots had little or no effect. The vehicle alone had minimal impact, confirming that the observed effects derive from the cDots itself. APBA–cDots produced a potent, dose-dependent inhibition of RSV infection (Fig. 2A and S6A). Even at moderate concentrations, APBA–cDots significantly reduced RSV infectivity and at the highest concentration tested virtually complete protection was observed. In contrast, unmodified CA–cDots showed only a marginal effect on RSV at any dose. A similar trend was observed for IAV. APBA–cDots suppressed CPE from both H1N1 and H3N2 strains in a dose-dependent manner (Fig. 2B and S6B). Statistically significant CPE reduction was achieved at mid-to-high concentrations of APBA–cDots, whereas CA–cDots again had negligible effect. APBA–cDots were likewise effective against hMPV infection (Fig. 2C and S6C). Both hMPV strains showed strongly reduced CPE and immunoplaque formation in the presence of APBA–cDots, in a concentration-dependent manner compared to CA–cDots. At the highest APBA–cDots dose tested, nearly full protection was observed for both hMPV strains, indicating potent neutralization. However, APBA–cDots did not show antiviral activity if the viruses were allowed to adsorb and bind to the cells followed by cDot treatment (Fig. S7), further supporting that viruses are neutralized only upon direct contact with cDots (*i.e.*, pre-exposure of viruses with cDots). Notably, pre-incubation of APBA–cDots with *N*-acetylglucosamine (NAG), a *cis*-diol-containing sugar, reduced their antiviral activity against IAV (Fig. S8). This competitive effect is consistent with partial occupation of boronic acid-containing surface sites by free sugar prior to virus exposure, thereby attenuating APBA–cDot-mediated viral inhibition. While not definitive on its own, these data provide direct experimental support for the involvement of boronic acid-mediated glycan recognition in the antiviral activity of APBA–cDots. Overall, APBA–cDots showed broad-spectrum antiviral efficacy against every tested respiratory virus.

To benchmark our APBA–cDots against an existing antiviral, we compared their efficacy to ribavirin, a broad-spectrum nucleoside analogue. Ribavirin was included at a standard reference concentration in parallel CPE assays.^21^ The antiviral efficacy of APBA–cDots was benchmarked against multiple doses of ribavirin in preceding trials (Fig. S9) as a positive assay control at a standard reference concentration to confirm assay validity. APBA–cDots demonstrated concentration-dependent CPE inhibition across all tested virus strains, consistent with meaningful antiviral activity in this system (Fig. S10 and S11). For RSV, APBA–cDots at a given concentration produced equal or greater CPE inhibition than ribavirin. The same trend held for IAV and hMPV. APBA–cDots achieved potent neutralization of each virus at doses, whereas ribavirin only partially suppressed infection. This suggests that APBA–cDots have at least the same intrinsic potency as ribavirin against these viruses, but with the added advantage of lower toxicity. Given that the clinical use of ribavirin is limited by host toxicity and marginal benefit, the finding that non-toxic APBA–cDots can achieve equivalent or better inhibition is highly encouraging. In summary, APBA–cDots performed as well as (or better than) the standard-of-care antiviral under identical *in vitro* conditions, underscoring the potential of APBA–cDots as practical therapeutic agents.

**Fig. 3:**
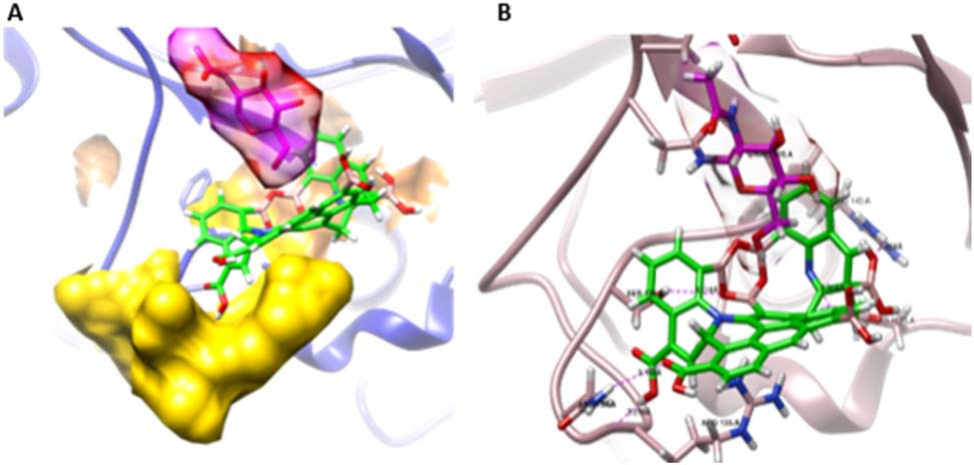
APBA–cDot selectively binds to RBS of IAV hemagglutinin (HA) glycoprotein. (A) Spatial localization of APBA–cDots with the IAV HA receptor binding site (RBS). The 130-loop (131–138, 140 residues) which is part of the RBS components is highlighted yellow. (B) Visualization of APBA–cDots (coloured green) interactions with 4WEA RBS residues *via* hydrogen bonds (pink dashed lines). NAG is coloured magenta.

To further investigate the role of glycan recognition in the antiviral activity of APBA–cDots against IAV, we performed molecular docking using NAG-containing IAV hemagglutinin (HA; PDB 4WEA) as a receptor model. Previous work has shown that a simplified APBA–cDot model specifically targeted the receptor binding sites (RBS) of the glycan-rich 4WEA.^22^ Consistent with the competitive inhibition observed in the NAG-blocking experiment (Fig. S8), APBA–cDots showed preferential localization near the HA receptor-binding site in the glycosylated model, whereas this selectivity was diminished in the glycan-depleted model (Fig. 3, S12–S15 and Tables S1, S2). Comparison with unmodified cDots and free APBA further showed that the APBA–cDot model exhibited higher binding affinity and stronger receptor binding site (RBS)-associated binding preference. Together, these findings support a model in which boronic acid-mediated glycan recognition contributes to APBA–cDot antiviral activity, while also suggesting that additional non-glycan interactions may be involved.

CDots have recently emerged as versatile antiviral agents, but almost all prior examples targeted viruses other than RSV or hMPV. Barras *et al.*^8^ showed that CDots prepared from 4-APBA block HSV-1 entry, whereas analogous CDots made from phenylboronic acid alone were completely inactive. Interestingly, carbonized glycyrrhizic acid cDots (GA-cDots) have been shown to potently inhibit IAV and protect mice from lethal challenge.^23^ We previously reported that APBA–cDots bind to the HIV-1 gp120 envelope protein to prevent viral entry.^10^ However, no previous cDot formulation has been shown to neutralize RSV or hMPV. Here, we show that APBA-functionalized cDots demonstrate potent neutralization of RSV, IAV and hMPV, a capability not previously reported. However, there are important caveats. All antiviral data were obtained *in vitro* using standard cell lines, which do not capture the multicellular architecture, immune cell milieu or other tissue-level features of the human respiratory tract. Thus, antiviral effects in these monolayer cultures may not predict performance in actual lung tissue. Critically, we did not perform any *in vivo* studies. Therefore, efficacy against infection in whole animals and any effects on biodistribution, pharmacokinetics or host immune response are unknown. In future work, we will address these gaps by testing APBA–cDots in relevant animal infection models, and conducting comprehensive *in vivo* toxicity and safety profiling. Concurrently, we will also explore formulation development (*e.g.*, nebulized or dry-powder aerosols) to enable efficient respiratory delivery and optimized lung deposition of these cDots.

This study presents preliminary *in vitro* evidence that APBA-functionalized carbon dots exhibit antiviral activity against a panel of respiratory viruses under laboratory cell culture conditions. While these findings are encouraging and support the potential of boronic acid-functionalized nanomaterials as a platform for antiviral development, they remain exploratory in nature. The results require validation in more physiologically relevant models, including three-dimensional airway epithelial cultures and *in vivo* infection systems, before conclusions regarding broad-spectrum therapeutic efficacy can be drawn. Mechanistically, the combined pre-exposure dependence, NAG competition assay, and molecular docking analyses support the interpretation that boronic acid-mediated glycan recognition contributes to viral neutralization. However, the present data do not exclude additional surface interactions, and therefore the mechanism should be regarded as strongly supported rather than definitively established. Nonetheless, this work provides a rationale for further mechanistic and translational investigation of glycan-targeted carbon nanomaterials.

## Conflicts of interest

Adi Idris is Director of Research at Intelligene Pty Ltd and holds shares in the company.

## Supplementary Material

NA-OLF-D6NA00007J-s001

## Data Availability

The authors confirm that the data supporting the findings of this study are available within the article and its supplementary information (SI). Supplementary information is available. See DOI: https://doi.org/10.1039/d6na00007j.
