# Supplementary material for: 2-Aminophenylboronic acid-functionalized carbon dots show broad-spectrum antiviral activity against respiratory viruses
Source: Nanoscale Adv. 2026 Jun 26. Online ahead of print. doi: 10.1039/d6na00007j (PMC13330788; doi:10.1039/d6na00007j)
Supplement: NA-OLF-D6NA00007J-s001 [file NA-OLF-D6NA00007J-s001.pdf]

Supplementary Information (SI) for Nanoscale Advances.
This journal is © The Royal Society of Chemistry 2026

Supplementary Information

2-aminophenylboronic acid-functionalized carbon dots show broad-spectrum antiviral activity against respiratory viruses

Musbahu Adam Ahmad[1#], Tufael Ahmed[2#], Mochamad Zakki Fahmi[1,3*], Adi Idris[2*]

[1]*Department of Chemistry, Airlangga University, Surabaya 60115, Indonesia*

[2]*Centre for Immunology and Infection Control, School of Biomedical Sciences, Queensland University of Technology, Kelvin Grove, QLD 4702, Australia*

[3]*Supra modification Nano-Micro Engineering Research Group, Airlangga University, Surabaya 60115, Indonesia*

***Corresponding authors:** a2.idris@qut.edu.au, Centre for Immunology and Infection Control, School of Biomedical Sciences, Queensland University of Technology, Brisbane, QLD, Australia

m.zakki.fahmi@fst.unair.ac.id, Supra modification Nano-Micro Engineering Research Group, Airlangga University, Surabaya 60115, Indonesia

[#]Authors share first authorship

[*]Authors share senior authorship

Supplementary Experimental Section

Materials and Method

Chemicals

Commercially available analytical grade reagents were used without further purifications. 2-aminophenylboronic acid (APBA, 97%), citric acid (CA, 99%), hydrochloric acid (HCl, 37%), and sodium hydroxide (NaOH, 97%) were purchased from Sigma-Aldrich (Milwaukee, WI, USA).

Cell culture

The Madin-Darby Canine Kidney (MDCK), Rhesus Monkey Kidney (Lilly Laboratories Culture – MK2) (LLC-MK2) and Human Epithelial type 2 (HEp-2) cell lines were obtained from the American Tissue Culture Collection (ATCC, VI, USA). All the *in vitro* stimulation assays were performed in Dulbecco's Modified Eagle Medium (DMEM) (Cytiva, Germany) containing varying concentrations (1-10%) of fetal bovine serum (FBS) (Cytiva, Germany), Penicillin (100 U/mL) and Streptomycin (100 µg/mL). All cells were maintained at 37°C in a humidified incubator with 5% CO_2.

Viruses

Influenza A (IAV) (H1N1 and H3N2 strains), respiratory syncytial viruses (RSV) (RSV-A2 and RSV4) and human metapneumovirus (hMPV) (AUS-001 and CAN-97-83 strains) were all grown and cultivated in MDCK, HEp-2 and LLC-MK2 cells, respectively. Professor Kirsten Spann (Queensland University of Technology) kindly provided all these virus strains. All viral stocks were concentrated and purified in Amicon Ultra-15 Centrifugal Filter units (Merck, Germany).

Synthesis of APBA-cDots

APBA-cDot was synthesized following the method we previously reported [1, 2]. Briefly, 216mg of CA as carbon source and 184mg of 2-APBA as a source of boron and nitrogen were mixed and subjected to pyrolysis at 270°C for 4h in a Thermolyne type 47900 furnace (Thermo Scientific, UK). After cooling to room temperature (RT), the black solid product was dissolved in 1M NaOH solution followed by sonication in a water bath (Power Sonic410). The pH of the solution was set to neutral followed by purification through a 0.22µm filter membrane.

Cdot characterization

Transmission electron microscopy (TEM) was performed using Talos L120C (ThermoFisher Scientific Inc.). Atomic force microscopy (AFM) images of the cDots were acquired using the scanning probe AFM5500M instrument (Hitachi, Japan). The Raman spectrum of the cDots were recorded using a T-64000 Horiba Jobin-Yvon LABRAMHR (Horiba, Japan) with a focal length of 800mm. The size diameter and zeta potential of the nanoparticles were determined with the Dynamic Light Scattering (DLS) instrument Delsa™ Nano HC Zeta Potential (Backman Coulter Inc., USA). CDot X-ray diffraction (XRD) and its spectrum were determined using a Rigaku Smart Lab X-ray diffractometer (Rigaku, Tokyo, Japan) with the Cu Kα 1 line (l = 1.54 Å). X-ray Photoelectron Spectroscopy (XPS) was conducted using AXIS Ultra DLD X-ray Photoelectron Spectroscopy Instrument (Shimadzu, Japan). Fourier transforms infrared (FTIR) spectra was recorded with an IRTracer-100 FTIR spectrophotometer (Shimadzu, Japan). UV-Visible spectral absorption was recorded on a Genesys 150 UV-vis spectrophotometer (Thermo Scientific, UK). The sample solution was scanned between the wavelength range of 200nm to 800nm. Photoluminescence (PL) spectra were measured using a BK-F96Pro Fluorescence Spectrophotometer equipped with xenon lamp (Biobase, China).

The Quantum Yield (QY) was calculated using the following equation:

$$\varphi_{APBA-cDots} = \varphi_{reference}\left(\frac{I_{APBA-cDots}}{I_{reference}}\right)\left(\frac{A_{APBA-cDots}}{A_{reference}}\right)\left(\frac{\eta_{water}}{\eta_{ethanol}}\right)^2$$

where:

Symbol	Definition
$\Phi_{\mathbf{APBA\text{ - }cDots}}$	Quantum yield of APBA-cDots (dimensionless, 0–1)
$\Phi_{\mathbf{ref}}$	Quantum yield of the reference fluorophore, Rhodamine 6G in ethanol = 0.95
$I_{\mathbf{APBA\text{ - }cDots}}$	Integrated area under the photoluminescence emission curve of APBA-cDots, obtained by integrating the

	full emission spectrum at a fixed excitation wavelength
$I_{\mathbf{ref}}$	Integrated area under the photoluminescence emission curve of Rhodamine 6G, measured under identical instrumental conditions
$A_{\mathbf{APBA\text{ - }cDots}}$	UV-visible absorbance of the APBA-cDot solution at the chosen excitation wavelength (kept below 0.1 to minimise inner filter effects)
$A_{\mathbf{ref}}$	UV-visible absorbance of the Rhodamine 6G solution at the same excitation wavelength (also kept below 0.1)
$\eta_{\mathbf{APBA\text{ - }cDots}}$	Refractive index of the solvent used for APBA-cDots (water, η= 1.333)
$\eta_{\mathbf{ref}}$	Refractive index of the solvent used for the reference (ethanol, η= 1.361)

Both APBA-cDot and Rhodamine 6G solutions were prepared at matched absorbance values (< 0.1 at the excitation wavelength) to satisfy the dilute-solution approximation and avoid reabsorption artefacts. All fluorescence measurements were conducted using the same instrument settings (slit width, detector sensitivity, and excitation wavelength) to ensure direct comparability of integrated emission intensities.

In vitro cytotoxicity assessment of cDots

cDots were dissolved separately in deionized water containing 0.1% DMSO (Merck, Germany), vortexed for 1 min, and sonicated using a probe sonicator at 10,000rpm for 3min. The suspensions were centrifuged at 10,000rpm for 10min and then filtered through a 0.22 µm filter. To assess the cytotoxicity of cDots on various cell lines, cDots dissolved in 0.1% DMSO in deionized water were diluted in culture media at desired concentrations. Confluent cells (96-well plates) were then treated with various concentrations of cDots over a 48h period. Following incubation, the medium was discarded, cells were washed with PBS, and 100 µL of 10% CCK-8 cell viability reagent (Vazyme, China) solution was added to each well. Colour development was observed within 15 min, and absorbance was recorded at 450 nm using a microplate reader.

In vitro antiviral efficacy assessment

To evaluate the neutralizing anti-viral effect of cDots *in vitro*, cDots at varying concentrations or vehicle control (0.1% DMSO) were co-incubated with 100 plaque forming units (pfu) of virus in cell media at room temperature for 30 mins with continuous rocking. Ribavirin (Cell signalling technology, MA, USA), a purine ribonucleoside analogue with broad-spectrum anti-viral properties against RNA viruses [3], was used as a positive control. After incubation, the cDot–virus mixtures were exposed to cells in 96-well plates. Following 7 days of incubation, cells were washed thoroughly (3x in PBS) before staining with 1% crystal violet solution (30mins). Crystal violet was then dissolved in 70% ethanol and absorbance measured at 600nm on a microplate reader. The cell viability was considered proportional to the amount of crystal violet retained and the absorbance value is expressed as a percentage. 100 – cell viability (%) = cytopathic effect (CPE, %), where a high viral infection correlates to higher CPE.

Determination of IC_{50} of APBA-cDot

APBA-cDots were serially diluted in infection medium containing 0.1% DMSO to final concentrations ranging from 10 – 0.0025 mg/ml. The APBA-cDots were pre-incubated with the three viral strains at room temperature for 30 mins with continuous rocking, followed by adding 100 pfu/well of virus particles on a 96 well plate pre-seeded with cell monolayers and incubated for 7 days to allow viral replication. Following incubation, the plates were washed with PBS and stained with crystal violet. The stain was subsequently solubilized in 70% ethanol and absorbance recorded at 600nm using a microplate reader and percentage of inhibition was calculated relative to untreated virus controls. The IC_{50} values were determined using the Graphpad prism v10 were the dose-response curves were fitted using nonlinear regression with 95% confidence interval and R^2 ranging from 0.923 - 0.961.

Inhibition of the antiviral activity of APBA-cDots using N-acetylglucosamine (NAG)

NAG was dissolved in PBS to make final concentrations of 1, 50 and 100 mM and filtered through a 0.22 µm syringe filter. The APBA or control cDots at a concentration of 10 mg/mL were incubated with NAG at room temperature for 1 hour to allow formation of covalent bonds between boronic acid and cis-diol. The resulting complexes were then pre-incubated with the virus strain for 30 minutes at room temperature prior to addition onto confluent cell monolayers seeded in 96-well plates. Inhibition of virus-induced cytopathic effect was quantified using crystal violet staining, and absorbance was measured at 600 nm using a microplate reader.

Receptor and Ligand Preparation

The crystal structure of IAV (H3N2) hemagglutinin (HA) was retrieved from the Protein Data Bank (PDB ID: 4WEA). Protein preparation was performed using the DockPrep tool in UCSF Chimera [4], involving the addition of missing hydrogens and assignment of AMBER ff14SB force field charges. For control simulations, N-acetyl-D-glucosamine (NAG) residues were manually deleted to generate the "NAG-removed" receptor model.

Based on the available experimental data, APBA-cDot and unmodified CA-cDots were developed to encompass most functional groups (**Figure S16**). The structure of the models and that of free APBA were optimized using Gaussian 16 software suite. Geometry optimization and frequency calculations were carried out at Becke, 3-parameter, LeeYang-Parr (B3LYP) level of theory. A split-basis approach was employed for the cDot models [5]. Briefly, 6-31-G(d) basis set was used for carbon and hydrogen atoms, while 6-31+G(d) basis set was assigned for boron, nitrogen and oxygen. To provide accurate parameters for subsequent molecular docking simulations, partial atomic charges were derived using the Restrained Electrostatic Potential (RESP) method with the aid of Multiwfn [6].

Docking Protocol

Blind docking simulations were executed using DOCK6 [7] to evaluate binding preferences across the entire HA surface without a predefined site bias. A grid-based scoring function was utilized with a grid spacing of 0.5 Å. The grid size was set to cover the whole receptor. To ensure exhaustive sampling of the conformational space, a maximum of 5,000 orientations were generated per ligand. Poses were ranked based on the Grid Score.

$$Grid\ score = E_{vdw} + E_{elec}$$

Where E_{vdw} is the Van der Waals energy component and E_{elec} is the electrostatics energy components.

Statistical analysis

All statistical analyses were performed using the statistical software package GraphPad Prism v10 and described in detail in respective figure legends. All data were represented by scatter plot and bar as mean ± SEM. Statistical significance between groups were determined by either one- or two-way ANOVA where appropriate with Tukey's post-hoc test.

Supplementary Tables

Table S1. Molecular docking energies of APBA-cDots and control ligands against IAV HA (PDB: 4WEA)

Receptor State	*Ligand*	*Grid Score (kcal/mol)*	*VdW Energy (kcal/mol)*	*Electrostatic (kcal/mol)*
4WEA	APBA-cDots	-57.1	-54.81	-2.29
	cDots	-55.92	-50.79	-5.13
	APBA	-28.32	-22.93	-5.39
NAG-removed 4WEA	APBA-cDots	-50.68	-46.62	-4.06
	cDots	-48.98	-45.27	-3.71
	APBA	-25.2	-20.86	-4.33

$$\boldsymbol{Grid\ score = E_{vdw} + E_{elec}}$$

Table S2. Hydrogen bond profiles of APBA-cDots and control ligands.

Receptor State	*Ligand*	*Primary Target*	*Key Residues*	*H-Bond Valency*
4WEA	APBA-cDots	RBS/130 Loop	ARG 143, SER 139, ASN 137, ARG 135	4 Bonds
	cDots	Peripheral Loop	GLN 73, ASN 126	2 Bonds

	APBA	Deep Internal Cleft	ARG 248, THR 128, GLY 127	4 Bonds
NAG-removed 4WEA	APBA-cDots	Distant Surface	LYS 442, GLU 432	3 Bonds
	cDots	Distant Surface	PHE 321, ASP 450	2 Bonds
	APBA	Distant Surface	GLU 432	1 Bond

Supplementary Figures

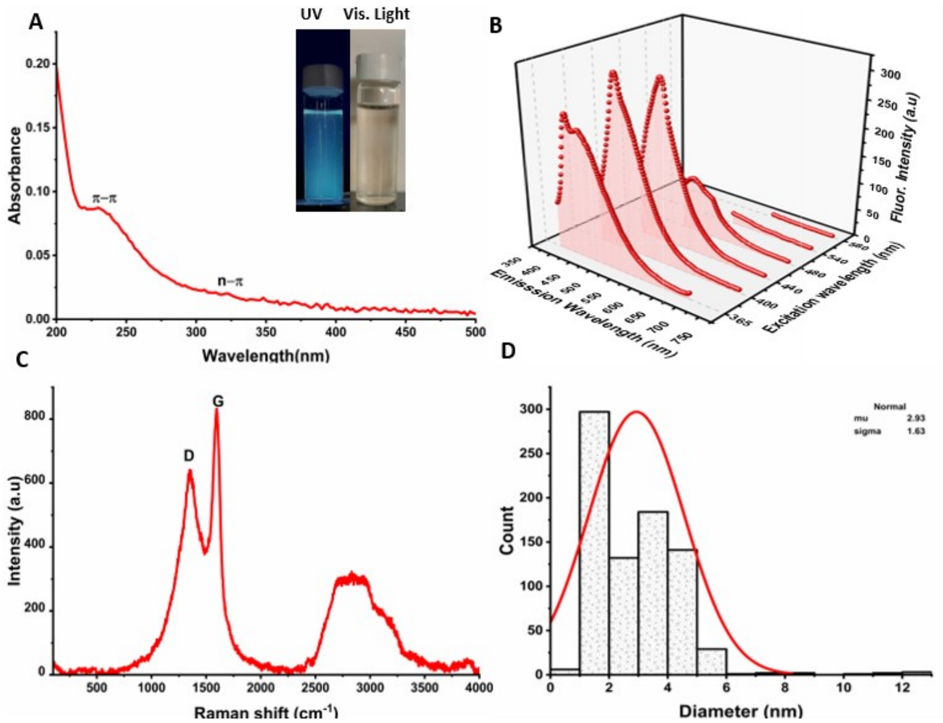

Figure S1 **Optical and structural characterisation of APBA-cDots.** (A) UV-visible absorption spectrum of APBA-cDots (50 µg/mL in deionised water). The absorption feature in the UV region is attributed to π→π* transitions of the sp^2 aromatic carbon skeleton and n→π* transitions arising from heteroatom (nitrogen, oxygen, boron) surface functionalities. Inset: photographs of APBA-cDot solution under visible light (left, pale yellow) and UV irradiation at 365 nm (right, blue-green fluorescence), visually confirming the photoluminescent properties of the synthesised cDots. (B) Photoluminescence (PL) emission spectra of APBA-cDots (50 µg/mL) recorded under sequential excitation wavelengths from 300 nm to 500 nm (increment as indicated). APBA-cDots exhibit a characteristic excitation-dependent emission pattern, with maximum emission intensity achieved under 400 nm excitation. This excitation-dependent behaviour is typical of carbon dots and is attributed to the distribution of surface emissive trap states arising from heterogeneous surface functionalities. Quantum yield (QY) was calculated relative to Rhodamine 6G as a reference standard using the comparative method. (C) Raman spectrum of APBA-cDots. Two prominent bands are observed: the D band at ~1359 cm^{-1}, associated with disordered sp^3 carbon and defect sites, and the G band at ~1596 cm^{-1}, associated with in-plane stretching of sp^2 graphitic carbon. The intensity ratio I_D/I_G = 0.77 indicates a significant degree of graphitisation with retained surface defects, consistent with a partially ordered graphitic core structure. Second-order Raman features (overtones and combination bands) are also visible in the 2500–3500 cm^{-1} region. (D) TEM particle size distribution histogram of APBA-cDots derived from analysis of >100 individual particles using ImageJ software. The distribution confirms a narrow size range centred at approximately 3 nm, consistent with the TEM micrograph shown in Figure 1C. The monodisperse size profile supports the homogeneity of the pyrolysis synthesis approach.

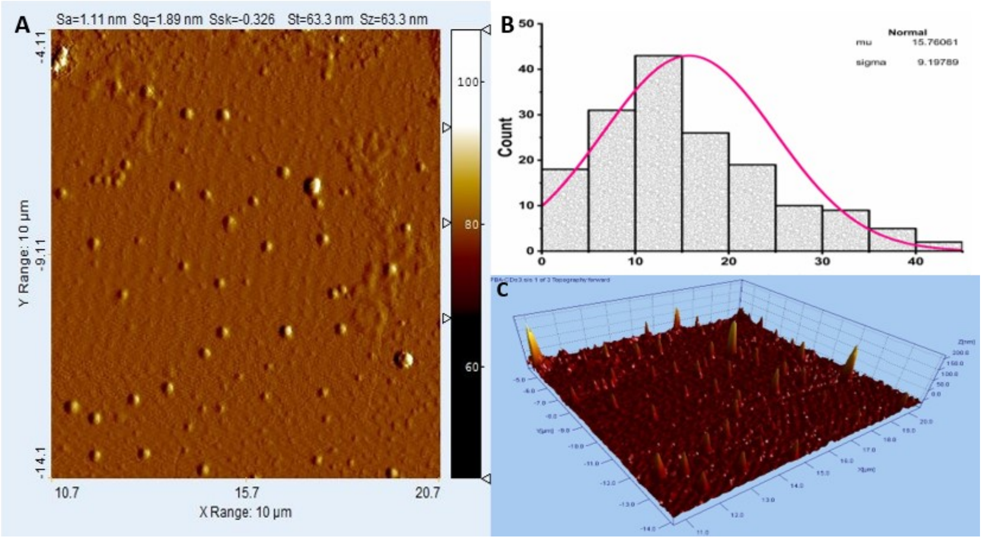

Figure S2 Atomic force microscopy (AFM) characterisation of APBA-cDots. (A) Two-dimensional (2D) AFM topographic image of APBA-cDots deposited on a mica substrate, acquired in tapping mode. Discrete, well-separated nanodot features are visible across the substrate surface, consistent with the particle morphology observed by TEM. (B) Height distribution histogram derived from AFM z-profile analysis of individual APBA-cDot particles. The average height is approximately 1.6 nm. The measured height values are notably larger than the TEM-derived lateral diameter (~3 nm), which is attributed to cluster formation and particle aggregation during sample drying and deposition on the mica substrate — a commonly reported artefact in AFM characterisation of carbon dots. Height measurements were analysed using ImageJ 1.54p. (C) Three-dimensional (3D) topographic AFM rendering of the same sample region shown in panel A, providing a visual representation of particle height distribution and surface topology. The 3D image further illustrates the discrete, dome-shaped profile of individual APBA-cDot clusters on the substrate surface.

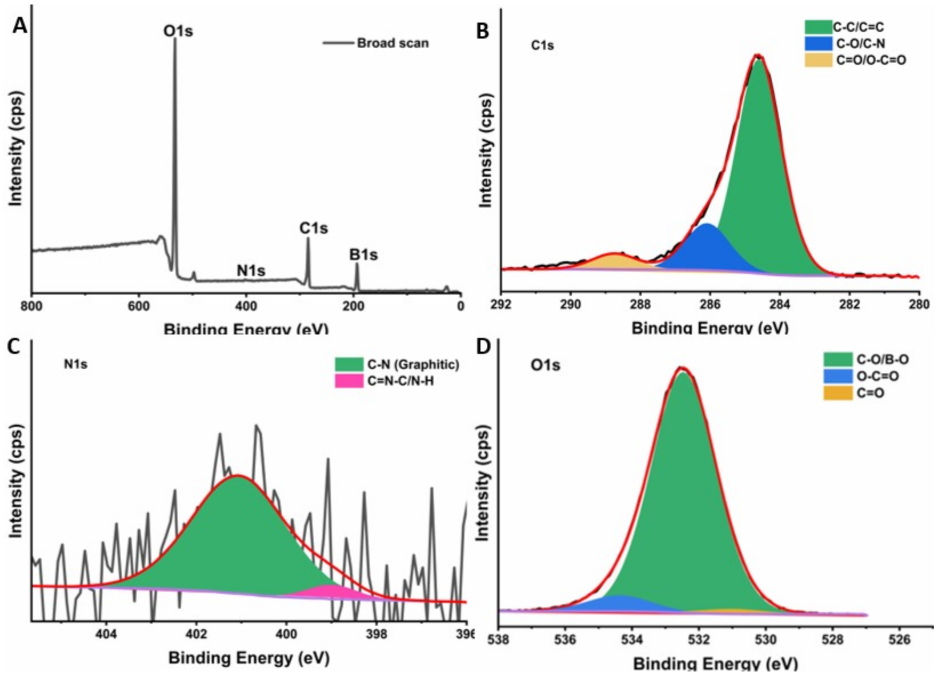

Figure S3 X-ray photoelectron spectroscopy (XPS) characterisation of APBA-cDots. (A) XPS broad survey spectrum of APBA-cDots, confirming the elemental composition of the nanoparticle surface. The survey spectrum identifies carbon (C 1s), oxygen (O 1s), nitrogen (N 1s), and boron (B 1s) as the principal surface elements, with boron and oxygen being particularly prominent, consistent with the boronic acid and boroxine-rich surface architecture of APBA-cDots. No significant impurity elements are detected. (B) High-resolution XPS spectrum of the C 1s region, deconvoluted into multiple sub-peaks corresponding to distinct carbon bonding environments: sp^2 graphitic carbon (C=C, ~284.6 eV), C–N/C–O bonds (~285.8–286.2 eV), C=O carbonyl (~287.5 eV), and O–C=O carboxyl (~288.8 eV). The presence of multiple carbon oxidation states confirms the chemically heterogeneous surface of the cDot core and is consistent with FTIR assignments. (C) High-resolution XPS spectrum of the N 1s region, deconvoluted to reveal nitrogen bonding environments arising from the APBA-derived amine functionality, including pyridinic N (~398.5 eV), pyrrolic/amino N (~399.8–400.2 eV), and graphitic N (~401.5 eV). The presence of nitrogen confirms successful incorporation of the amino group from the 2-aminophenylboronic acid precursor into the cDot surface during pyrolysis. (D) High-resolution XPS spectrum of the O 1s region, deconvoluted into contributions from C=O (~531.5 eV), C–OH/B–OH (~532.5 eV), and C–O–C (~533.5 eV) bonding environments. The prominent B–OH component at ~532.5 eV corroborates the FTIR and B 1s XPS data confirming abundant surface boronic acid groups available for cis-diol binding interactions.

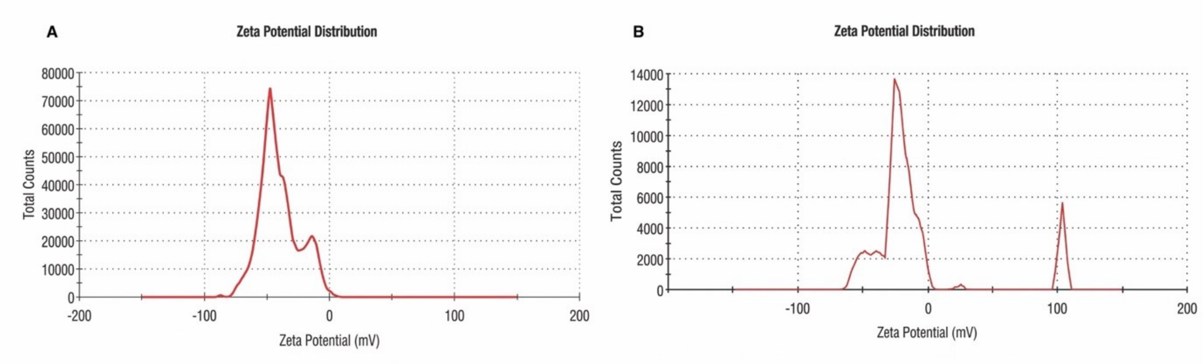

Figure S4 Zeta potential analysis of cDots. The results indicate a shift toward more positive values for APBA-cDots compared to pristine CA-cDots. Zeta potential of (A) carboxylate- and hydroxyl-rich CA-cDots (−55.6 mV) and (B) nitrogen- and boron-containing APBA-cDots (−10.0 mV). Measurements were performed in aqueous dispersion at room temperature. Data are shown as mean values from replicate determinations.

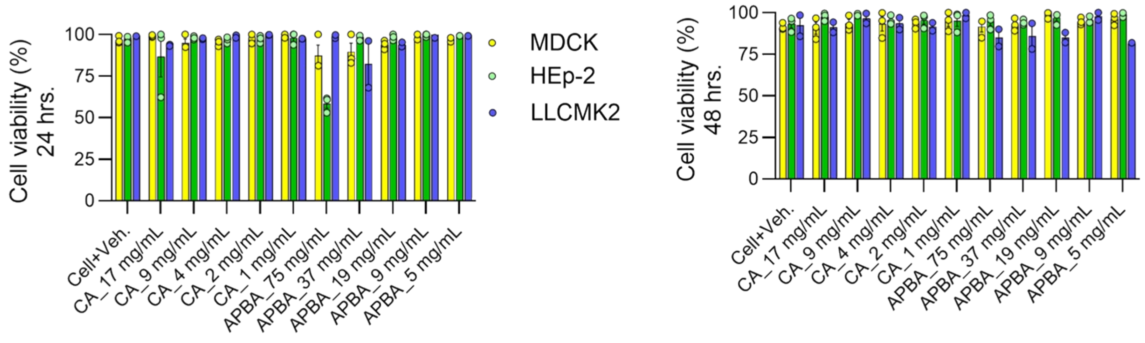

Figure S5. APBA-cDots are not cytotoxic. MDCK, LLC-MK2 and HEp-2 cell were incubated with the indicated doses of either APBA-modified cDots (APBA) or unmodified cDots (CA) for 24 and 48 hrs before measuring cell viability using the CCK-8 assay. 0.1% DMSO diluted in cell media serves as the vehicle control. Data are represented by scatter plot and bar as mean ± SEM. Statistical significance between different groups was determined using two-way ANOVA with Tukey's post-hoc test and * denotes p < 0.05, ** denotes p < 0.01 and *** denotes p < 0.001.

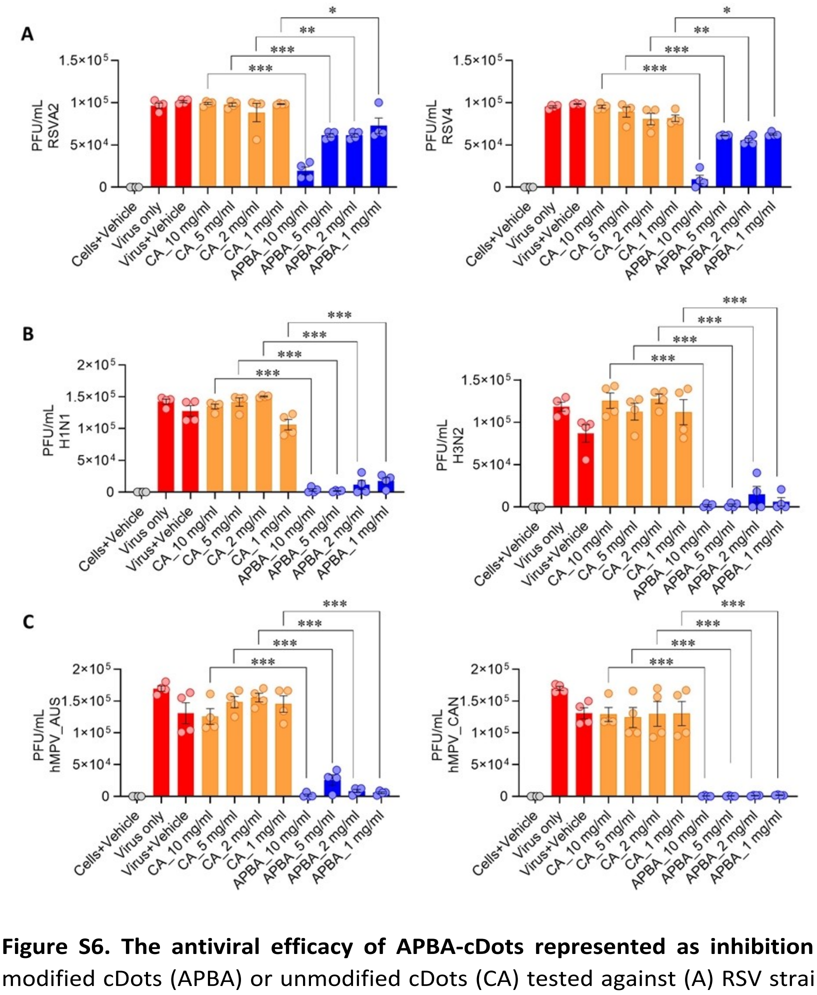

Figure S6. The antiviral efficacy of APBA-cDots represented as inhibition of immunoplaques *in vitro*. APBA-modified cDots (APBA) or unmodified cDots (CA) tested against (A) RSV strains RSV-A2 (left) and RSV4 (right), (B) IAV strain H1N1 (left) and H3N2 (right) and (C) HMPV strains AUS-001 (AUS) (left) and CAN-97-83. Data are represented by scatter plot and bar as mean ± SEM. Statistical significance between different groups was determined using one-way ANOVA with Tukey's post-hoc test and * denotes p < 0.05, ** denotes p < 0.01 and *** denotes p < 0.001.

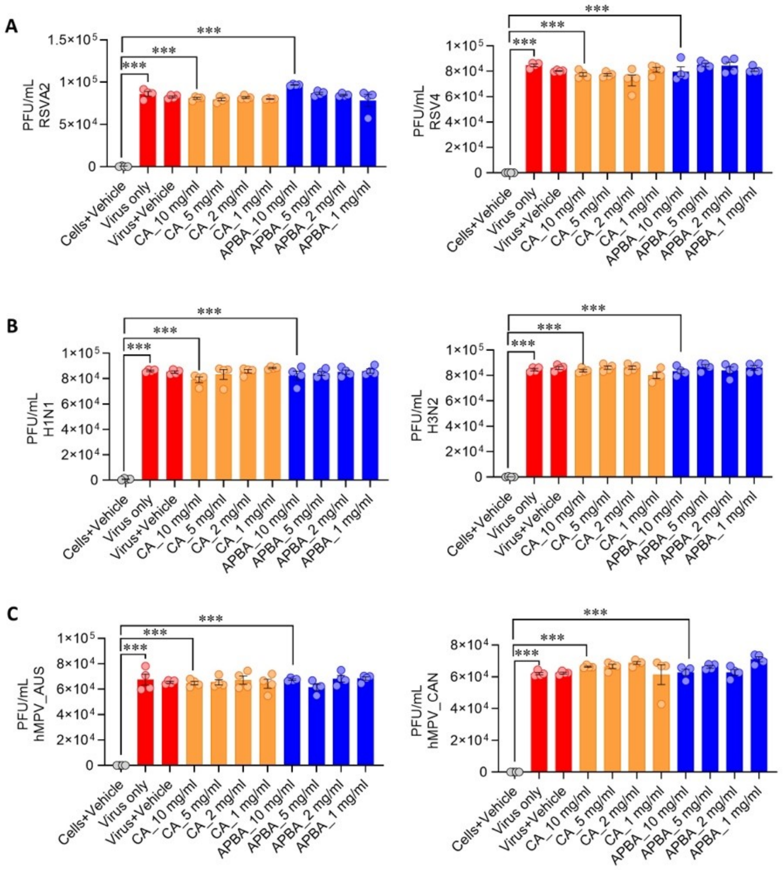

Figure S7. APBA-cDots do not show antiviral activity post infection *in vitro*. Different cell lines were incubated with viruses (100 PFU/well) and incubated at 37 °C for 2hr for virus adsorption. Following incubation, the virus containing media was removed and cells were incubated with in media containing APBA-modified cDots (APBA) or unmodified cDots (CA) and plaque counts were measured 7 days post infection. Figure shows plaque counts of (A) RSV strains RSV-A2 (left) and RSV4 (right), (B) IAV strain H1N1 (left) and H3N2 (right) and (C) HMPV strains AUS-001 (AUS) (left) and CAN-97-83. Data are represented by scatter plot and bar as mean ± SEM. Statistical significance between different groups was determined using one-way ANOVA with Tukey's post-hoc test and * denotes $p < 0.05$, ** denotes $p < 0.01$ and *** denotes $p < 0.001$.

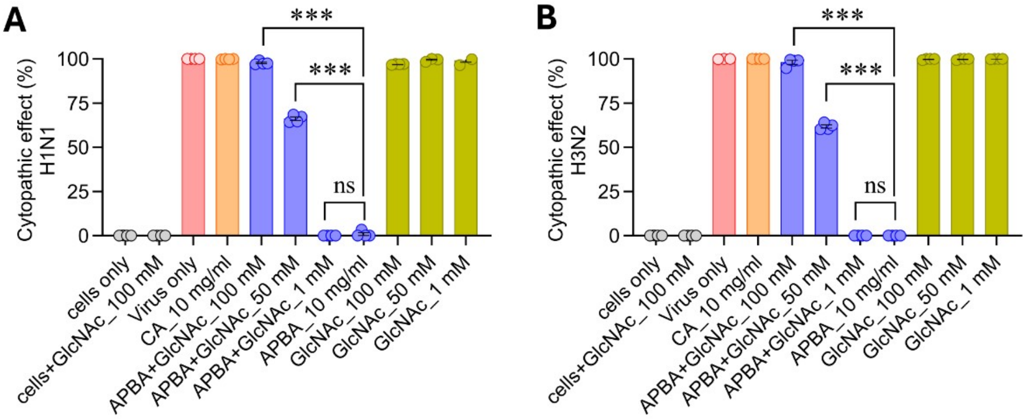

Figure S8. N-acetylglucosamine (NAG) inhibited the antiviral activity of APBA-cDots. APBA-cDots were pre-incubated with increasing concentrations of NAG, a cis-diol-containing monosaccharide, prior to exposure to influenza A virus (IAV). Results are shown as mean ± SEM from replicate measurements. Statistical significance between groups was determined by one-way ANOVA with Tukey's post-hoc test; * denotes $p < 0.05$, ** denotes $p < 0.01$, and *** denotes $p < 0.001$.

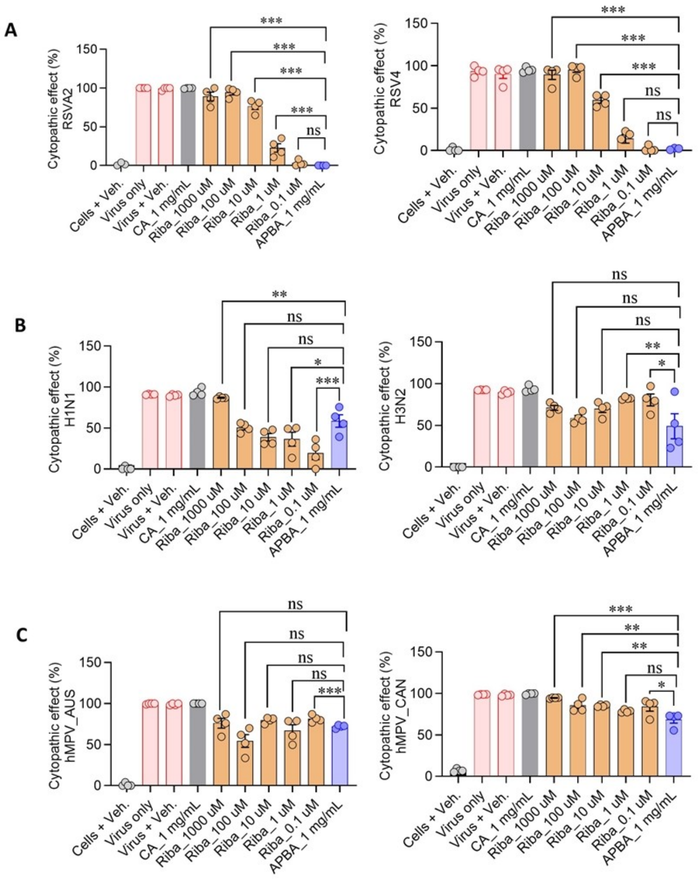

Figure S9. The antiviral efficacy of APBA-cDots compared to different doses standard-of-care antiviral drug, ribavirin *in vitro*. Anti-viral activity of either APBA-modified cDots (APBA), unmodified cDots (CA) or ribavirin (Riba) at the indicated concentrations represented as inhibition of virus-mediated cytopathic effect (CPE) against a range of viruses (A) RSV strains RSV-A2 (left) and RSV4 (right); (B) IAV strain H1N1 (left) and H3N2 (right); (C) HMPV strains AUS-001 (AUS) (left) and CAN-97-83 (CAN) (right). 0.1% DMSO diluted in cell media serves as the vehicle control. Data are represented by scatter plot and bar as mean ± SEM. Statistical significance between different groups was determined using one-way ANOVA with Tukey's post-hoc test and * denotes $p < 0.05$, ** denotes $p < 0.01$ and *** denotes $p < 0.001$.

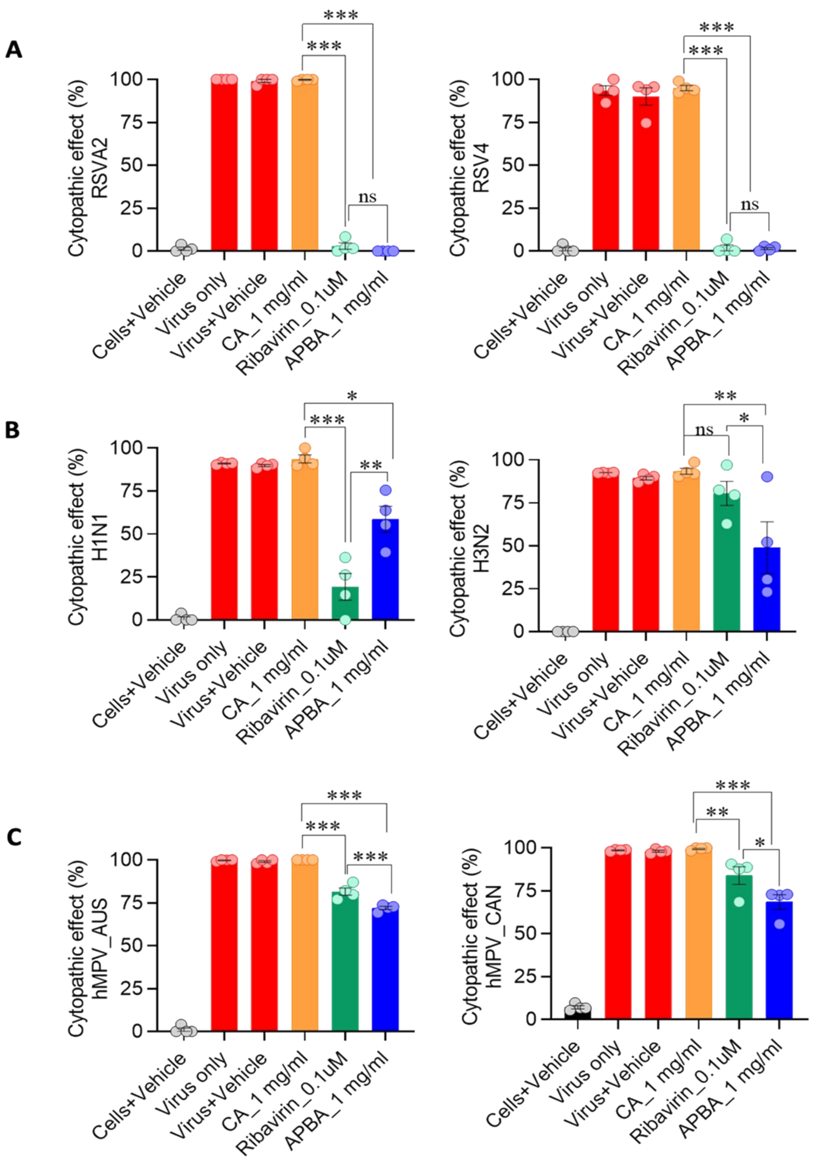

Figure S10. The antiviral efficacy of APBA-cDots is comparable or better than the standard-of-care antiviral drug, ribavirin *in vitro*. Anti-viral activity of either APBA-modified cDots (APBA), unmodified cDots (CA) or ribavirin at the indicated concentrations represented as inhibition of virus-mediated cytopathic effect (CPE) against a range of viruses (A) RSV strains RSV-A2 (left) and RSV4 (right); (B) IAV strain H1N1 (left) and H3N2 (right); (C) HMPV strains AUS-001 (AUS) (left) and CAN-97-83 (CAN) (right). 0.1% DMSO diluted in cell media serves as the vehicle control. Data are represented by scatter plot and bar as mean ± SEM. Statistical significance between different groups was determined using one-way ANOVA with Tukey's post-hoc test and * denotes $p < 0.05$, ** denotes $p < 0.01$ and *** denotes $p < 0.001$.

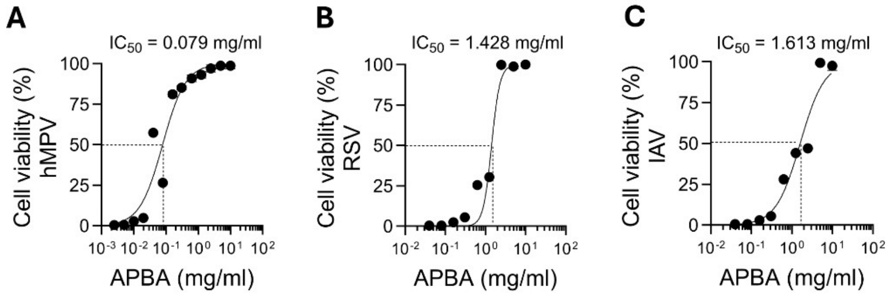

Figure S11. APBA-cDots are antivirally potent in a dose-dependent manner *in vitro*. APBA-functionalized carbon dots (APBA-cDots) were tested at increasing concentrations in pre-exposure antiviral assays against the indicated respiratory viruses. Virus was co-incubated with APBA-cDots before addition to the appropriate host cells, and antiviral activity was measured as inhibition of virus-induced cytopathic effect (CPE) after 7 days of incubation period. Data are shown as mean scatter plots.

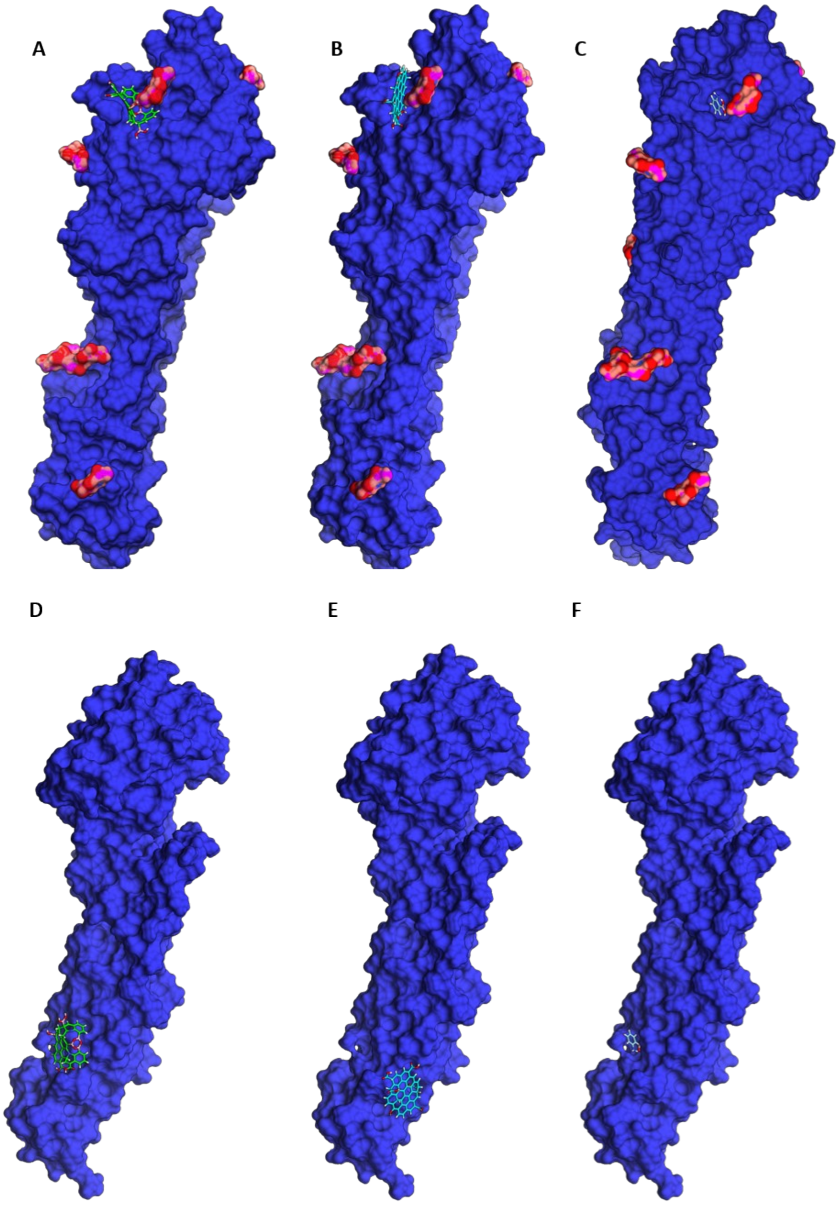

Figure S12 Global site-selectivity and topographical localization of ligands on IAV hemagglutinin (HA) receptor. (A–C) Global binding orientations of (A) APBA-cDots, localized specifically at the functional RBS interface, sequestering the 130-loop (residues 131–138, 140) and the 144-flank. (B) unmodified cDots, and (C) free APBA, which are sequestered at potentially non-functional peripheral loops (e.g., Asn126) or recessed internal clefts (e.g., Arg248), on the native HA globular head (PDB: 4WEA). The magenta surface denotes the N-acetylglucosamine (NAG) residue. **(D–F)** Comparative orientations on the NAG-depleted receptor, demonstrating the disruption of glycan-guided homing and the subsequent migration of ligands to distant, non-functional surface regions (e.g., Lys442, Glu432).

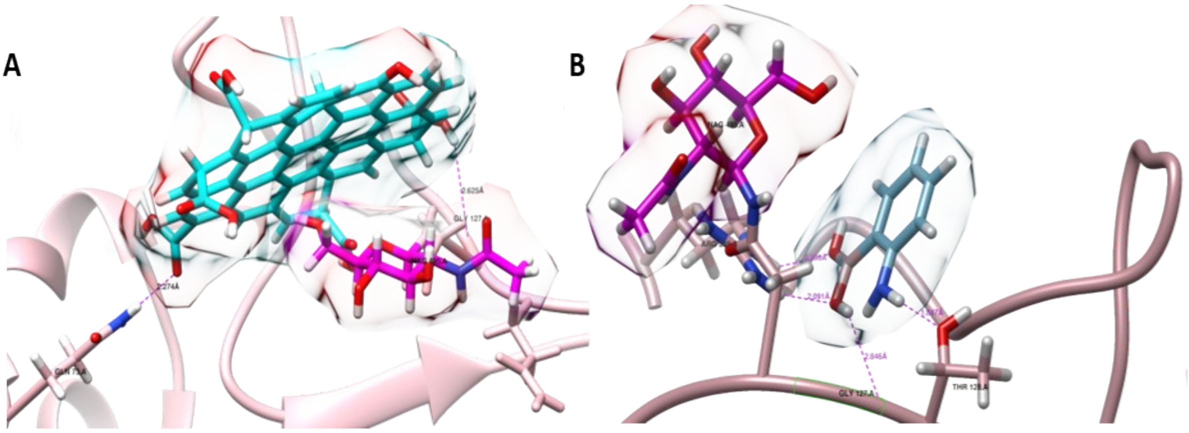

Figure S13 Interaction profiles on native HA (4WEA). (A) unmodified cDots exhibit low-valency surface adsorption at GLN 73 and ASN 126. (B) Free APBA is sequestered within a deep internal cleft involving THR 128 and ARG 248.

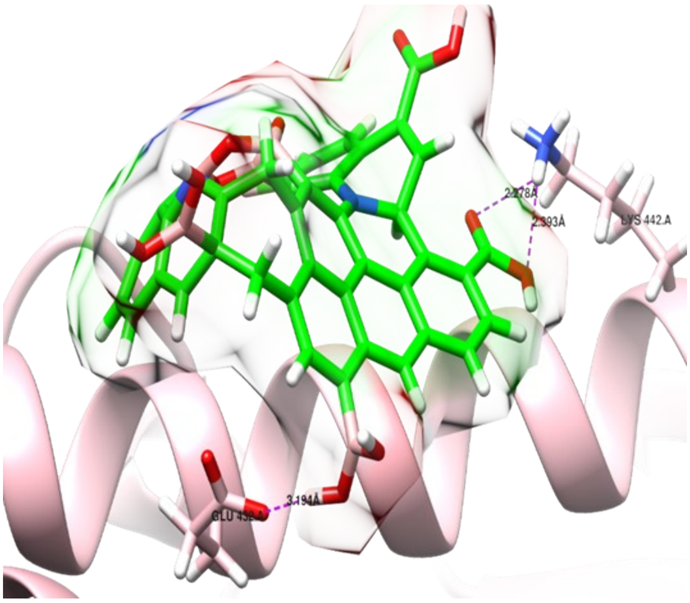

Figure S14 Docking of APBA-cDots against NAG-removed 4WEA demonstrates complete migration from the RBS to a distant surface defined by LYS 442 and GLU 432. The resulting binding energy difference (ΔE = 6.42 kcal/mol) confirms that boronic acid functionalization acts as a specific sensor for evolved glycosylation sites (e.g., Asn144) flanking the 130-loop.

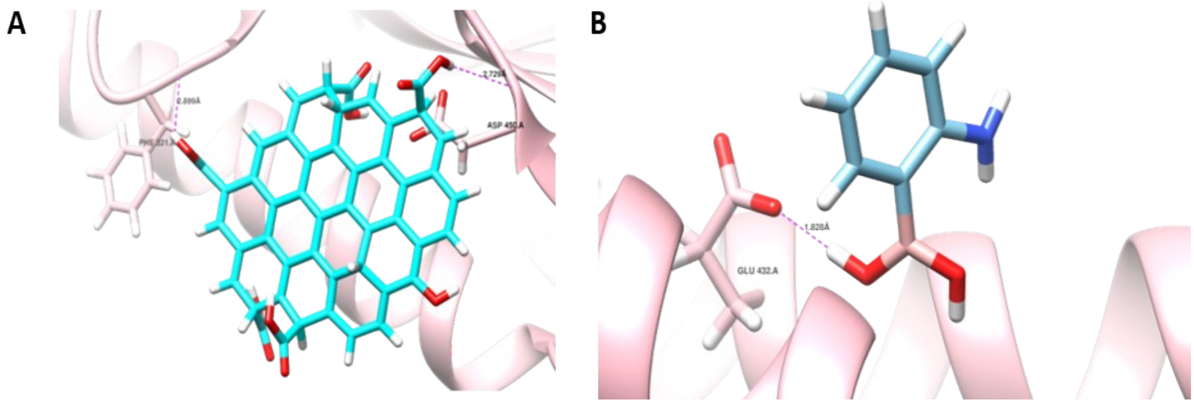

Figure S15 Topographical migration upon glycan depletion resulted. Hydrogen-bonding analysis on NAG-removed 4WEA for both (A) unmodified cDots and (B) free APBA proved that the absence of the N-acetylglucosamine (NAG) spatial signal causes ligands to exhibit stochastic migration to potentially non-functional surface regions, including PHE 321 and GLU 432.

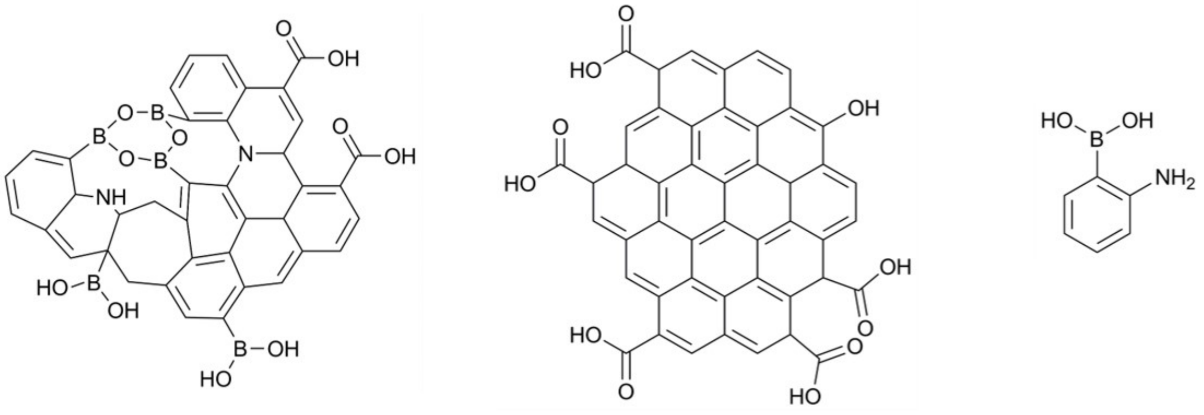

APBA-cDot **CA-cDot** **APBA**

Figure S16 Chemical structures of simple APBA-cDots, CA-cDots and APBA models.

References (supplementary section)

1. Y. Y. Aung, A. N. Kristanti, S. Q. Khairunisa, N. Nasronudin and M. Z. Fahmi, *ACS Biomaterials Science & Engineering*, 2020, **6**, 4490-4501.
2. A. Wibrianto, Y. J. Saputra, S. F. A. Sugito, S. Q. Khairunisa, B. E. Rachman, N. Nasronudin, N. L. A. Megasari, J. Y. Chang and M. Z. Fahmi, *J Pharm Biomed Anal*, 2024, **248**, 116242.
3. S. Crotty, D. Maag, J. J. Arnold, W. Zhong, J. Y. Lau, Z. Hong, R. Andino and C. E. Cameron, *Nat Med*, 2000, **6**, 1375-1379.
4. E. F. Pettersen, T. D. Goddard, C. C. Huang, G. S. Couch, D. M. Greenblatt, E. C. Meng and T. E. Ferrin, *J Comput Chem*, 2004, **25**, 1605-1612.
5. J. Chen, F. Li, J. Yang, J. Gu, K. Ellerbe, B. Zhao, M. Shiri, Y. Zhou, A. Ajith, A. Joji, W. Zhang, D. Abayawardena, A. H. Wikramanayake, K. Wang, F. Zhang, F. Verde, R. Prabhakar, Y. Zhang and R. M. Leblanc, *Journal of Advanced Research*, 2025, DOI: https://doi.org/10.1016/j.jare.2025.11.004.
6. T. Lu, *J Chem Phys*, 2024, **161**.
7. J. D. Bickel, B. T. Boysan and R. C. Rizzo, *Journal of Computational Chemistry*, 2025, **46**, e27508.
